# 15-Meth­oxy-14,15-di­hydro­andranginine

**DOI:** 10.1107/S1600536813011604

**Published:** 2013-05-04

**Authors:** Dian-Lei Wang, Xiang-Hai Cai, Peng Huang, He-Ping Huang

**Affiliations:** a School of Pharmacy, Anhui University of Traditional Chinese Medicine, Hefei 230038, People’s Republic of China; b State Key Laboratory of Phytochemistry and Plant Resources in West China, Kunming Institute of Botany, Chinese Academy of Sciences, Kunming 650204, People’s Republic of China

## Abstract

The title polycyclic alkaloid, C_22_H_26_N_2_O_3_, an indole derivative obtained from *Melodinus yunnanensis*, comprises three chiral C atoms and crystallizes as a racemate. Its seven-membered heterocyclic ring has a twisted conformation, with the N atom within the plane of the indole moiety and with two adjacent C atoms deviating in opposite directions from its plane by 0.756 (3) (methyl­ene C) and −0.802 (3) Å (methine C). In the crystal, pairs of N—H⋯O hydrogen bonds connect the mol­ecules into centrosymmetric dimers.

## Related literature

Indole alkaloid derivatives obtained from *Melodinus yunnanensis* have been investigated due to their anti­malarial and anti­cancer properties, see: Kanfan *et al.* (1974 ▶). For the structures of related compounds, see: Danieli *et al.* (1977*a* ▶) and for applications of similar compounds see: Danieli *et al.* (1977*a* ▶,*b* ▶)
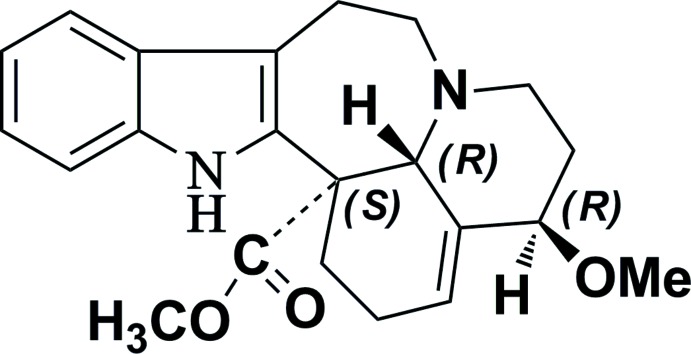


## Experimental

### 

#### Crystal data

C_22_H_26_N_2_O_3_
*M* *_r_* = 366.45
Triclinic, 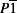

*a* = 6.914 (3) Å
*b* = 11.232 (4) Å
*c* = 11.806 (4) Å
α = 91.079 (6)°
β = 100.737 (5)°
γ = 96.317 (6)°
*V* = 894.5 (6) Å^3^
*Z* = 2
Mo *K*α radiation
μ = 0.09 mm^−1^
*T* = 153 K
0.43 × 0.23 × 0.13 mm

#### Data collection

Rigaku AFC10/Saturn724+ diffractometer
8672 measured reflections
4028 independent reflections
2868 reflections with *I* > 2σ(*I*)
*R*_int_ = 0.030

#### Refinement

*R*[*F* ^2^ > 2σ(*F* ^2^)] = 0.041
*wR*(*F* ^2^) = 0.089
*S* = 1.00
4028 reflections
250 parameters
H atoms treated by a mixture of independent and constrained refinement
Δρ_max_ = 0.27 e Å^−3^
Δρ_min_ = −0.19 e Å^−3^

### 

Data collection: *CrystalClear* (Rigaku, 2008 ▶); cell refinement: *CrystalClear*; data reduction: *CrystalClear*; program(s) used to solve structure: *SHELXS97* (Sheldrick, 2008 ▶); program(s) used to refine structure: *SHELXL97* (Sheldrick, 2008 ▶); molecular graphics: *SHELXTL* (Sheldrick, 2008 ▶); software used to prepare material for publication: *SHELXTL*.

## Supplementary Material

Crystal structure: contains datablock(s) I, global. DOI: 10.1107/S1600536813011604/ld2092sup1.cif

Structure factors: contains datablock(s) I. DOI: 10.1107/S1600536813011604/ld2092Isup2.hkl

Additional supplementary materials:  crystallographic information; 3D view; checkCIF report

## Figures and Tables

**Table 1:** Hydrogen-bond geometry (Å, °)

*D*—H⋯*A*	*D*—H	H⋯*A*	*D*⋯*A*	*D*—H⋯*A*
N1—H1*N*⋯O3^i^	0.859 (17)	2.187 (16)	2.9759 (18)	152.6 (15)

Symmetry code: (i) 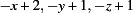.

**Fig. 1.**
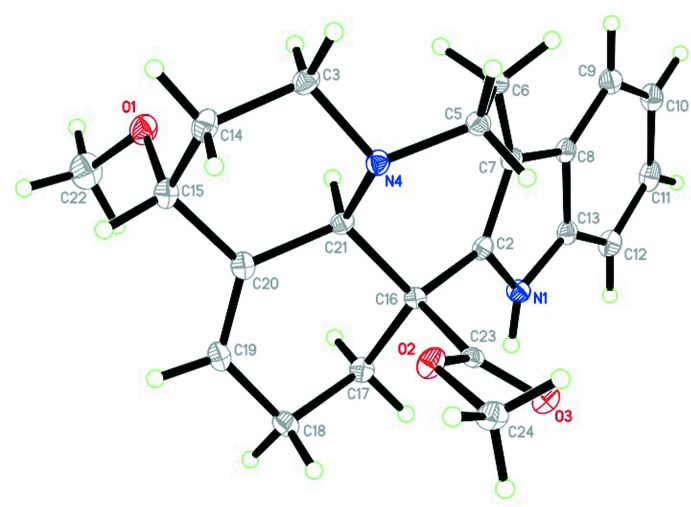
: The molecular structure of the title molecule(I) with atom labels and 30% probability displacement ellipsoids.

**Fig. 2.**
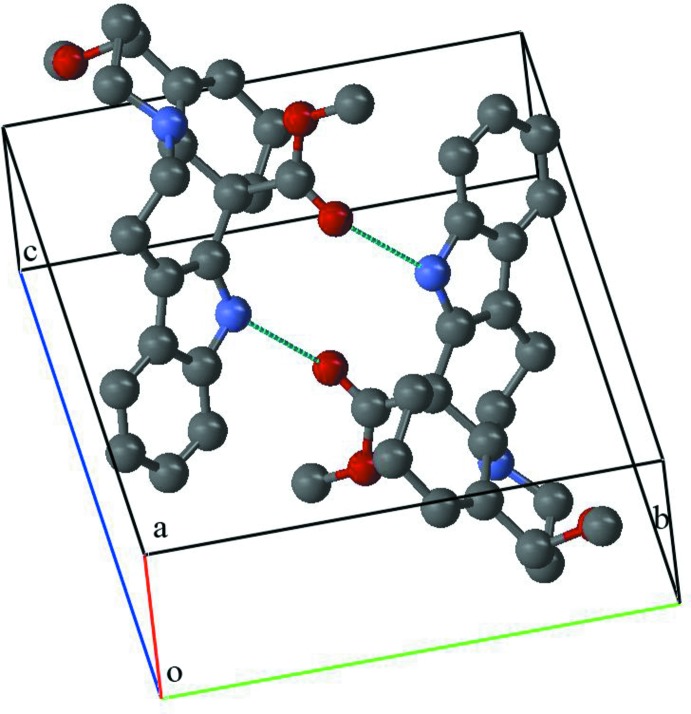
: packing diagram of (I). Hydrogen bonds shown as dashed lines.

## supplementary crystallographic information

### Comment

The indole alkaloid derivatives of the title compound, (I), are obtained from Melodinus yunnanensis leaves and twigs by column chromatography. These indole alkaloid derivatives have been investigated due to their antimalarial and anticancer properties (Kanfan *et al.* 1974). Herewith we present the crystal structure of (I).

### Experimental

Dried and powdered leaves and twigs of Melodinus yunnanensis (20 kg) were extracted three times with methanol at room temperature and the solvent evaporated *in vacuo*. The residue was dissolved in 0.3% aqueous hydrochloric acid, and the solution subsequently basified using ammonia water to pH 9–10. The basic solution was partitioned with EtOAc, producing an aqueous and EtOAc phase. The resulting EtOAc fraction (105 g) was collected and then subjected to column chromatography over silica gel and eluted with a chloroform-acetone gradient (1/0 to 3/1, *v*/*v*) to afford the title compound (500 mg). The product was purified by recrystalliaztion from methanol to give colorless crystals.

### Refinement

All hydrogen atoms were placed in geometrically idealized positions and constrained to ride on their parent atoms, with C—H = 0.95-1.00 Å and *U*_iso_(H) = 1.2 *U*_eq_. Positions of hydrogen atoms of methyl groups were rotationally optimized. Atom H of amino group was refined isotropically without restrictions.

### Crystal data

**Table:**

C_22_H_26_N_2_O_3_	*Z* = 2
*M**_r_* = 366.45	*F*(000) = 392
Triclinic, *P*1	*D*_x_ = 1.361 Mg m^−^^3^
Hall symbol: -P 1	Mo *K*α radiation, λ = 0.71073 Å
*a* = 6.914 (3) Å	Cell parameters from 2653 reflections
*b* = 11.232 (4) Å	θ = 3.0–27.5°
*c* = 11.806 (4) Å	µ = 0.09 mm^−^^1^
α = 91.079 (6)°	*T* = 153 K
β = 100.737 (5)°	Prism, colorless
γ = 96.317 (6)°	0.43 × 0.23 × 0.13 mm
*V* = 894.5 (6) Å^3^	

### Data collection

**Table:**

Rigaku AFC10/Saturn724+ diffractometer	2868 reflections with *I* > 2σ(*I*)
Radiation source: Rotating Anode	*R*_int_ = 0.030
Graphite monochromator	θ_max_ = 27.5°, θ_min_ = 3.0°
Detector resolution: 28.5714 pixels mm^-1^	*h* = −8→8
phi and ω scans	*k* = −14→14
8672 measured reflections	*l* = −14→15
4028 independent reflections	

### Refinement

**Table:**

Refinement on *F*^2^	Primary atom site location: structure-invariant direct methods
Least-squares matrix: full	Secondary atom site location: difference Fourier map
*R*[*F*^2^ > 2σ(*F*^2^)] = 0.041	Hydrogen site location: inferred from neighbouring sites
*wR*(*F*^2^) = 0.089	H atoms treated by a mixture of independent and constrained refinement
*S* = 1.00	*w* = 1/[σ^2^(*F*_o_^2^) + (0.0318*P*)^2^ + 0.160*P*] where *P* = (*F*_o_^2^ + 2*F*_c_^2^)/3
4028 reflections	(Δ/σ)_max_ = 0.001
250 parameters	Δρ_max_ = 0.27 e Å^−^^3^
0 restraints	Δρ_min_ = −0.19 e Å^−^^3^

### Special details

**Table:**

Geometry. All e.s.d.'s (except the e.s.d. in the dihedral angle between two l.s. planes) are estimated using the full covariance matrix. The cell e.s.d.'s are taken into account individually in the estimation of e.s.d.'s in distances, angles and torsion angles; correlations between e.s.d.'s in cell parameters are only used when they are defined by crystal symmetry. An approximate (isotropic) treatment of cell e.s.d.'s is used for estimating e.s.d.'s involving l.s. planes.
Refinement. Refinement of *F*^2^ against ALL reflections. The weighted *R*-factor *wR* and goodness of fit *S* are based on *F*^2^, conventional *R*-factors *R* are based on *F*, with *F* set to zero for negative *F*^2^. The threshold expression of *F*^2^ > σ(*F*^2^) is used only for calculating *R*-factors(gt) *etc*. and is not relevant to the choice of reflections for refinement. *R*-factors based on *F*^2^ are statistically about twice as large as those based on *F*, and *R*- factors based on ALL data will be even larger.

### Fractional atomic coordinates and isotropic or equivalent isotropic displacement parameters (Å^2^)

**Table:**

	*x*	*y*	*z*	*U*_iso_*/*U*_eq_	
O1	0.60683 (16)	0.85999 (9)	0.01190 (9)	0.0277 (3)	
O2	0.46541 (15)	0.48350 (9)	0.27560 (8)	0.0239 (2)	
O3	0.67895 (15)	0.46849 (9)	0.44057 (8)	0.0244 (2)	
N1	1.00622 (18)	0.69322 (12)	0.50300 (10)	0.0205 (3)	
N4	0.42553 (17)	0.72717 (10)	0.24809 (9)	0.0185 (3)	
C2	0.8300 (2)	0.71262 (13)	0.43152 (11)	0.0175 (3)	
C3	0.3346 (2)	0.81220 (14)	0.16762 (12)	0.0229 (3)	
H3A	0.4208	0.8895	0.1758	0.028*	
H3B	0.2048	0.8269	0.1856	0.028*	
C5	0.4122 (2)	0.75869 (13)	0.36721 (11)	0.0194 (3)	
H5A	0.4215	0.6854	0.4123	0.023*	
H5B	0.2793	0.7840	0.3669	0.023*	
C6	0.5671 (2)	0.85711 (13)	0.43018 (12)	0.0215 (3)	
H6A	0.5876	0.9223	0.3769	0.026*	
H6B	0.5176	0.8911	0.4959	0.026*	
C7	0.7607 (2)	0.81020 (13)	0.47397 (12)	0.0189 (3)	
C8	0.8993 (2)	0.85448 (13)	0.57542 (12)	0.0201 (3)	
C9	0.9085 (2)	0.95044 (14)	0.65452 (12)	0.0246 (3)	
H9	0.8069	1.0019	0.6457	0.030*	
C10	1.0671 (2)	0.96896 (14)	0.74531 (13)	0.0275 (4)	
H10	1.0739	1.0336	0.7996	0.033*	
C11	1.2176 (2)	0.89460 (14)	0.75890 (13)	0.0280 (4)	
H11	1.3261	0.9102	0.8217	0.034*	
C12	1.2125 (2)	0.79874 (14)	0.68306 (12)	0.0262 (4)	
H12	1.3149	0.7478	0.6929	0.031*	
C13	1.0515 (2)	0.77946 (13)	0.59135 (12)	0.0202 (3)	
C14	0.3065 (2)	0.76125 (15)	0.04513 (12)	0.0259 (4)	
H14A	0.2199	0.6841	0.0371	0.031*	
H14B	0.2414	0.8173	−0.0092	0.031*	
C15	0.5046 (2)	0.74198 (14)	0.01609 (12)	0.0235 (3)	
H15	0.4846	0.6994	−0.0611	0.028*	
C16	0.7451 (2)	0.63502 (12)	0.32228 (11)	0.0168 (3)	
C17	0.9184 (2)	0.59056 (13)	0.27308 (12)	0.0214 (3)	
H17A	1.0106	0.6608	0.2605	0.026*	
H17B	0.9920	0.5411	0.3308	0.026*	
C18	0.8514 (2)	0.51737 (14)	0.16040 (12)	0.0242 (3)	
H18A	0.9685	0.5019	0.1276	0.029*	
H18B	0.7812	0.4392	0.1750	0.029*	
C19	0.7168 (2)	0.58305 (13)	0.07633 (12)	0.0231 (3)	
H19	0.6987	0.5606	−0.0033	0.028*	
C20	0.6212 (2)	0.67091 (13)	0.10661 (12)	0.0200 (3)	
C21	0.6292 (2)	0.71353 (12)	0.23085 (11)	0.0177 (3)	
H21	0.7039	0.7957	0.2404	0.021*	
C22	0.7679 (3)	0.86269 (17)	−0.04602 (15)	0.0386 (4)	
H22A	0.8327	0.9450	−0.0440	0.046*	
H22B	0.7199	0.8342	−0.1264	0.046*	
H22C	0.8632	0.8107	−0.0079	0.046*	
C23	0.6256 (2)	0.52135 (12)	0.35387 (12)	0.0174 (3)	
C24	0.3361 (2)	0.38405 (13)	0.30607 (13)	0.0250 (3)	
H24A	0.2227	0.3645	0.2427	0.030*	
H24B	0.2888	0.4061	0.3760	0.030*	
H24C	0.4092	0.3142	0.3202	0.030*	
H1N	1.078 (2)	0.6370 (16)	0.4959 (14)	0.032 (5)*	

### Atomic displacement parameters (Å^2^)

**Table:**

	*U*^11^	*U*^22^	*U*^33^	*U*^12^	*U*^13^	*U*^23^
O1	0.0297 (6)	0.0267 (6)	0.0277 (6)	0.0001 (5)	0.0091 (5)	0.0057 (5)
O2	0.0229 (6)	0.0209 (6)	0.0248 (6)	−0.0055 (4)	0.0007 (5)	0.0038 (4)
O3	0.0259 (6)	0.0219 (6)	0.0249 (6)	0.0035 (4)	0.0028 (5)	0.0062 (4)
N1	0.0180 (7)	0.0223 (7)	0.0209 (7)	0.0044 (5)	0.0020 (5)	0.0000 (5)
N4	0.0168 (6)	0.0219 (7)	0.0172 (6)	0.0036 (5)	0.0031 (5)	0.0027 (5)
C2	0.0161 (7)	0.0182 (7)	0.0174 (7)	−0.0001 (6)	0.0020 (6)	0.0035 (6)
C3	0.0193 (8)	0.0253 (8)	0.0249 (8)	0.0061 (6)	0.0035 (6)	0.0064 (6)
C5	0.0181 (7)	0.0198 (8)	0.0211 (7)	0.0036 (6)	0.0052 (6)	0.0018 (6)
C6	0.0225 (8)	0.0188 (8)	0.0232 (8)	0.0039 (6)	0.0036 (6)	−0.0007 (6)
C7	0.0196 (7)	0.0170 (7)	0.0194 (7)	0.0000 (6)	0.0032 (6)	0.0018 (6)
C8	0.0215 (8)	0.0205 (8)	0.0175 (7)	−0.0021 (6)	0.0039 (6)	0.0021 (6)
C9	0.0278 (9)	0.0212 (8)	0.0243 (8)	−0.0013 (6)	0.0063 (7)	−0.0001 (6)
C10	0.0338 (9)	0.0250 (8)	0.0213 (8)	−0.0062 (7)	0.0050 (7)	−0.0028 (6)
C11	0.0268 (9)	0.0341 (9)	0.0183 (8)	−0.0078 (7)	−0.0016 (7)	0.0023 (7)
C12	0.0217 (8)	0.0311 (9)	0.0244 (8)	0.0006 (7)	0.0017 (7)	0.0042 (7)
C13	0.0203 (8)	0.0209 (8)	0.0188 (7)	−0.0016 (6)	0.0045 (6)	0.0015 (6)
C14	0.0218 (8)	0.0328 (9)	0.0216 (8)	0.0025 (7)	0.0003 (6)	0.0063 (7)
C15	0.0237 (8)	0.0266 (8)	0.0190 (7)	−0.0003 (6)	0.0030 (6)	0.0014 (6)
C16	0.0164 (7)	0.0161 (7)	0.0176 (7)	0.0021 (6)	0.0030 (6)	0.0007 (5)
C17	0.0178 (7)	0.0238 (8)	0.0226 (8)	0.0022 (6)	0.0038 (6)	0.0012 (6)
C18	0.0245 (8)	0.0246 (8)	0.0250 (8)	0.0036 (6)	0.0084 (7)	−0.0023 (6)
C19	0.0245 (8)	0.0267 (8)	0.0178 (7)	−0.0008 (6)	0.0054 (6)	−0.0023 (6)
C20	0.0174 (7)	0.0223 (8)	0.0190 (7)	−0.0031 (6)	0.0035 (6)	0.0002 (6)
C21	0.0169 (7)	0.0160 (7)	0.0199 (7)	0.0009 (6)	0.0033 (6)	0.0021 (6)
C22	0.0347 (10)	0.0414 (11)	0.0410 (10)	−0.0059 (8)	0.0162 (8)	0.0048 (8)
C23	0.0176 (7)	0.0160 (7)	0.0200 (7)	0.0048 (6)	0.0061 (6)	−0.0011 (6)
C24	0.0223 (8)	0.0193 (8)	0.0332 (9)	−0.0031 (6)	0.0083 (7)	0.0009 (6)

### Geometric parameters (Å, º)

**Table:**

O1—C22	1.4091 (19)	C11—C12	1.381 (2)
O1—C15	1.4381 (18)	C11—H11	0.9500
O2—C23	1.3274 (17)	C12—C13	1.396 (2)
O2—C24	1.4461 (17)	C12—H12	0.9500
O3—C23	1.2109 (16)	C14—C15	1.509 (2)
N1—C13	1.3775 (19)	C14—H14A	0.9900
N1—C2	1.3881 (18)	C14—H14B	0.9900
N1—H1N	0.859 (17)	C15—C20	1.5105 (19)
N4—C5	1.4657 (18)	C15—H15	1.0000
N4—C3	1.4675 (17)	C16—C23	1.5337 (19)
N4—C21	1.4836 (18)	C16—C17	1.549 (2)
C2—C7	1.366 (2)	C16—C21	1.5665 (18)
C2—C16	1.5265 (19)	C17—C18	1.518 (2)
C3—C14	1.515 (2)	C17—H17A	0.9900
C3—H3A	0.9900	C17—H17B	0.9900
C3—H3B	0.9900	C18—C19	1.493 (2)
C5—C6	1.527 (2)	C18—H18A	0.9900
C5—H5A	0.9900	C18—H18B	0.9900
C5—H5B	0.9900	C19—C20	1.325 (2)
C6—C7	1.498 (2)	C19—H19	0.9500
C6—H6A	0.9900	C20—C21	1.523 (2)
C6—H6B	0.9900	C21—H21	1.0000
C7—C8	1.430 (2)	C22—H22A	0.9800
C8—C9	1.401 (2)	C22—H22B	0.9800
C8—C13	1.407 (2)	C22—H22C	0.9800
C9—C10	1.378 (2)	C24—H24A	0.9800
C9—H9	0.9500	C24—H24B	0.9800
C10—C11	1.393 (2)	C24—H24C	0.9800
C10—H10	0.9500		
			
C22—O1—C15	113.67 (12)	C3—C14—H14B	109.7
C23—O2—C24	116.57 (11)	H14A—C14—H14B	108.2
C13—N1—C2	109.27 (12)	O1—C15—C14	105.53 (12)
C13—N1—H1N	123.4 (11)	O1—C15—C20	110.41 (12)
C2—N1—H1N	127.3 (11)	C14—C15—C20	111.29 (12)
C5—N4—C3	111.16 (11)	O1—C15—H15	109.8
C5—N4—C21	114.27 (10)	C14—C15—H15	109.8
C3—N4—C21	110.72 (11)	C20—C15—H15	109.8
C7—C2—N1	108.82 (12)	C2—C16—C23	109.26 (11)
C7—C2—C16	129.33 (12)	C2—C16—C17	108.93 (11)
N1—C2—C16	121.80 (12)	C23—C16—C17	105.34 (11)
N4—C3—C14	109.50 (12)	C2—C16—C21	108.70 (11)
N4—C3—H3A	109.8	C23—C16—C21	115.68 (11)
C14—C3—H3A	109.8	C17—C16—C21	108.74 (11)
N4—C3—H3B	109.8	C18—C17—C16	113.48 (12)
C14—C3—H3B	109.8	C18—C17—H17A	108.9
H3A—C3—H3B	108.2	C16—C17—H17A	108.9
N4—C5—C6	116.69 (12)	C18—C17—H17B	108.9
N4—C5—H5A	108.1	C16—C17—H17B	108.9
C6—C5—H5A	108.1	H17A—C17—H17B	107.7
N4—C5—H5B	108.1	C19—C18—C17	110.25 (12)
C6—C5—H5B	108.1	C19—C18—H18A	109.6
H5A—C5—H5B	107.3	C17—C18—H18A	109.6
C7—C6—C5	111.61 (12)	C19—C18—H18B	109.6
C7—C6—H6A	109.3	C17—C18—H18B	109.6
C5—C6—H6A	109.3	H18A—C18—H18B	108.1
C7—C6—H6B	109.3	C20—C19—C18	123.64 (13)
C5—C6—H6B	109.3	C20—C19—H19	118.2
H6A—C6—H6B	108.0	C18—C19—H19	118.2
C2—C7—C8	107.44 (12)	C19—C20—C15	120.65 (13)
C2—C7—C6	127.05 (13)	C19—C20—C21	124.21 (13)
C8—C7—C6	125.29 (13)	C15—C20—C21	115.03 (12)
C9—C8—C13	119.11 (13)	N4—C21—C20	109.21 (11)
C9—C8—C7	133.72 (14)	N4—C21—C16	113.89 (11)
C13—C8—C7	107.18 (13)	C20—C21—C16	113.54 (11)
C10—C9—C8	118.96 (15)	N4—C21—H21	106.6
C10—C9—H9	120.5	C20—C21—H21	106.6
C8—C9—H9	120.5	C16—C21—H21	106.6
C9—C10—C11	121.12 (15)	O1—C22—H22A	109.5
C9—C10—H10	119.4	O1—C22—H22B	109.5
C11—C10—H10	119.4	H22A—C22—H22B	109.5
C12—C11—C10	121.44 (14)	O1—C22—H22C	109.5
C12—C11—H11	119.3	H22A—C22—H22C	109.5
C10—C11—H11	119.3	H22B—C22—H22C	109.5
C11—C12—C13	117.53 (15)	O3—C23—O2	123.37 (13)
C11—C12—H12	121.2	O3—C23—C16	122.35 (12)
C13—C12—H12	121.2	O2—C23—C16	114.20 (11)
N1—C13—C12	130.87 (14)	O2—C24—H24A	109.5
N1—C13—C8	107.29 (12)	O2—C24—H24B	109.5
C12—C13—C8	121.84 (14)	H24A—C24—H24B	109.5
C15—C14—C3	109.99 (12)	O2—C24—H24C	109.5
C15—C14—H14A	109.7	H24A—C24—H24C	109.5
C3—C14—H14A	109.7	H24B—C24—H24C	109.5
C15—C14—H14B	109.7		
			
C13—N1—C2—C7	−0.53 (16)	C7—C2—C16—C17	146.69 (14)
C13—N1—C2—C16	177.13 (12)	N1—C2—C16—C17	−30.45 (17)
C5—N4—C3—C14	−166.45 (12)	C7—C2—C16—C21	28.35 (19)
C21—N4—C3—C14	65.41 (15)	N1—C2—C16—C21	−148.79 (12)
C3—N4—C5—C6	−81.17 (15)	C2—C16—C17—C18	−177.04 (11)
C21—N4—C5—C6	45.04 (16)	C23—C16—C17—C18	65.86 (14)
N4—C5—C6—C7	−79.41 (15)	C21—C16—C17—C18	−58.73 (15)
N1—C2—C7—C8	0.47 (15)	C16—C17—C18—C19	50.95 (16)
C16—C2—C7—C8	−176.97 (13)	C17—C18—C19—C20	−19.8 (2)
N1—C2—C7—C6	−174.43 (13)	C18—C19—C20—C15	173.52 (13)
C16—C2—C7—C6	8.1 (2)	C18—C19—C20—C21	−2.4 (2)
C5—C6—C7—C2	24.9 (2)	O1—C15—C20—C19	−106.75 (16)
C5—C6—C7—C8	−149.13 (13)	C14—C15—C20—C19	136.40 (15)
C2—C7—C8—C9	180.00 (15)	O1—C15—C20—C21	69.54 (15)
C6—C7—C8—C9	−5.0 (2)	C14—C15—C20—C21	−47.31 (17)
C2—C7—C8—C13	−0.24 (15)	C5—N4—C21—C20	175.36 (11)
C6—C7—C8—C13	174.77 (13)	C3—N4—C21—C20	−58.20 (14)
C13—C8—C9—C10	0.4 (2)	C5—N4—C21—C16	47.27 (15)
C7—C8—C9—C10	−179.88 (15)	C3—N4—C21—C16	173.71 (11)
C8—C9—C10—C11	0.4 (2)	C19—C20—C21—N4	−134.12 (14)
C9—C10—C11—C12	−0.9 (2)	C15—C20—C21—N4	49.74 (15)
C10—C11—C12—C13	0.5 (2)	C19—C20—C21—C16	−5.84 (19)
C2—N1—C13—C12	−179.14 (14)	C15—C20—C21—C16	178.02 (12)
C2—N1—C13—C8	0.37 (15)	C2—C16—C21—N4	−81.24 (14)
C11—C12—C13—N1	179.70 (14)	C23—C16—C21—N4	42.06 (16)
C11—C12—C13—C8	0.3 (2)	C17—C16—C21—N4	160.30 (12)
C9—C8—C13—N1	179.72 (12)	C2—C16—C21—C20	152.92 (11)
C7—C8—C13—N1	−0.08 (15)	C23—C16—C21—C20	−83.78 (14)
C9—C8—C13—C12	−0.7 (2)	C17—C16—C21—C20	34.46 (15)
C7—C8—C13—C12	179.48 (13)	C24—O2—C23—O3	9.3 (2)
N4—C3—C14—C15	−61.07 (16)	C24—O2—C23—C16	−173.83 (12)
C22—O1—C15—C14	−162.46 (12)	C2—C16—C23—O3	−39.04 (18)
C22—O1—C15—C20	77.17 (15)	C17—C16—C23—O3	77.84 (16)
C3—C14—C15—O1	−68.38 (14)	C21—C16—C23—O3	−162.05 (12)
C3—C14—C15—C20	51.41 (17)	C2—C16—C23—O2	144.01 (12)
C7—C2—C16—C23	−98.72 (16)	C17—C16—C23—O2	−99.11 (13)
N1—C2—C16—C23	84.13 (16)	C21—C16—C23—O2	20.99 (17)

### Hydrogen-bond geometry (Å, º)

**Table:**

*D*—H···*A*	*D*—H	H···*A*	*D*···*A*	*D*—H···*A*
N1—H1*N*···O3^i^	0.859 (17)	2.187 (16)	2.9759 (18)	152.6 (15)

Symmetry code: (i) −*x*+2, −*y*+1, −*z*+1.
